# Spliceosomal gene mutations in myelodysplasia: molecular links to clonal abnormalities of hematopoiesis

**DOI:** 10.1101/gad.278424.116

**Published:** 2016-05-01

**Authors:** Daichi Inoue, Robert K. Bradley, Omar Abdel-Wahab

**Affiliations:** 1 Human Oncology and Pathogenesis Program, Department of Medicine, Memorial Sloan Kettering Cancer Center, New York, New York 10065, USA;; 2 Computational Biology Program, Public Health Sciences Division, Fred Hutchinson Cancer Research Center, Seattle, Washington 98109, USA;; 3 Basic Sciences Division, Fred Hutchinson Cancer Research Center, Seattle, Washington 98109, USA;; 4 Leukemia Service, Department of Medicine, Memorial Sloan Kettering Cancer Center, New York, New York 10065, USA

**Keywords:** leukemia, SF3B1, SRSF2, U2AF1, mRNA splicing

## Abstract

Genomic analyses of the myeloid malignancies and clonal disorders of hematopoiesis that may give rise to these disorders have identified that mutations in genes encoding core spliceosomal proteins and accessory regulatory splicing factors are among the most common targets of somatic mutations. These spliceosomal mutations often occur in a mutually exclusive manner with one another and, in aggregate, account for the most frequent class of mutations in patients with myelodysplastic syndromes (MDSs) in particular. Although substantial progress has been made in understanding the effects of several of these mutations on splicing and splice site recognition, functional connections linking the mechanistic changes in splicing induced by these mutations to the phenotypic consequences of clonal and aberrant hematopoiesis are not yet well defined. This review describes our current understanding of the mechanistic and biological effects of spliceosomal gene mutations in MDSs as well as the regulation of splicing throughout normal hematopoiesis.

## Abstract (precis)

Genomic analyses of myeloid malignancies have identified that mutations in genes encoding core spliceosomal proteins and accessory regulatory splicing factors are among the most common targets of somatic mutations. In this review, Inoue et al. describe our current understanding of the mechanistic and biological effects of spliceosomal gene mutations in myelodysplastic syndromes as well as the regulation of splicing throughout normal hematopoiesis.

Genomic analyses of the myeloid malignancies as well as the clonal disorders of hematopoiesis that may precede them have consistently identified that these conditions are enriched for mutations in genes encoding epigenetic regulatory proteins as well as proteins involved in mRNA splicing (Papaemmanuil et al. 2013; Haferlach et al. 2014; Jaiswal et al. 2014; Genovese et al. 2015). Since the initial discovery of spliceosomal gene mutations associated with hematological malignancies in 2011, it is now known that spliceosomal genes represent the most common targets of mutations in patients with myelodysplastic syndromes (MDSs), with these mutations occurring in ∼60%–70% of patients (Papaemmanuil et al. 2011; Yoshida et al. 2011; Graubert et al. 2012). Long known for being essential for producing mature mRNA molecules, splicing is also now widely understood to be a cotranscriptional and post-transcriptional mechanism essential for the regulation of gene expression in addition to modifying gene product function. Thus, alterations in RNA splicing caused by spliceosomal mutations add to the general transcriptional dysfunction that characterizes MDSs and related myeloid malignancies. Although much has been learned about the functional and therapeutic implications of epigenetic alterations in myeloid malignancy pathogenesis, understanding the functional roles of spliceosomal gene mutations is just beginning.

The discovery of spliceosomal mutations has raised an array of mechanistic, functional, and biological questions. The high frequencies of spliceosomal gene mutations as well as the evidence that many spliceosomal mutations confer an alteration of function rather than loss of function make understanding their functional effects critical. In this review, we describe what is currently known regarding mRNA splicing regulation in normal hematopoiesis as well as in the disordered clonal hematopoiesis characteristic of myeloid malignancies.

## Splicing in normal hematopoiesis

Our current understanding of normal hematopoiesis and aberrancies in hematopoietic differentiation resulting in leukemias derives mostly from knowledge of the transcription factors that direct lineage commitment and post-translational modifications that alter protein function. It is estimated that >90% of multiexon human genes undergo alternative splicing (AS) to generate proteome diversity (Wang et al. 2008), yet relatively little is known about the regulation and functional roles of AS in hematopoiesis. Basic mechanisms of splicing regulation have been detailed in several recent reviews (Fu and Ares 2014; Lee and Rio 2015).

**Figure 1.**
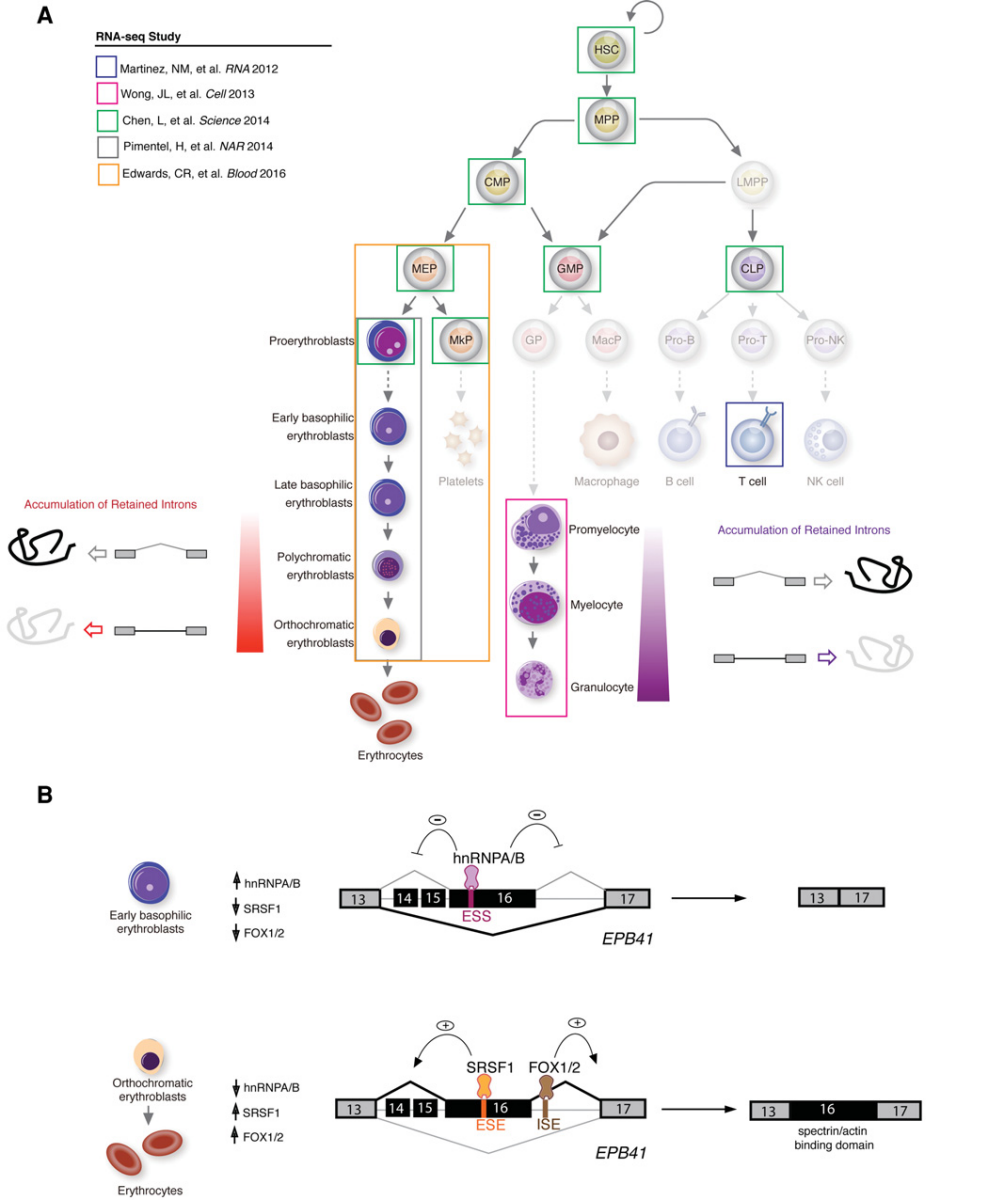
RNA-seq studies of hematopoiesis reveal stage-specific regulation of splicing during normal hematopoiesis. (*A*) Summary of RNA-seq studies of normal hematopoiesis performed to date with examples of models proposed regulating terminal erythropoiesis and late-stage granulopoiesis. Cell populations from mice or humans that have yet to be subjected to splicing analysis by RNA-seq analysis are opaquely shaded. (*B*) Examples of differentiation stage-specific splicing of *EPB41*, which encodes protein 4.1R, a vital component of the red blood cell membrane cytoskeleton. Exon 16, which encodes a portion of the spectrin/actin-binding domain, is skipped in early erythroid progenitors but included in mature erythroblasts (exons 14–15 are not thought to be expressed in hematopoietic cells and are largely restricted to expression in the brain [Conboy et al. 1991; Huang et al. 1993]). A series of studies has elucidated the *cis* elements and *trans*-acting splicing factors that orchestrate the stage-specific splicing of exon 16, as shown. Normally occurring alterations in expression of the RNA-binding proteins hnRNPA/B, SRSF1, and FOX1/2 throughout erythropoiesis are responsible for the exon 16 splicing switch. This occurs via inhibition of splicing by hnRNPA/B binding to an exon splicing silencer (ESS) (Hou et al. 2002) as well as promotion of exon inclusion by SRSF1 (Yang et al. 2005) and FOX1/2 (Deguillien et al. 2001; Ponthier et al. 2006) when they bind to exonic splicing enhancers (ESEs) and intronic splicing enhancers (ISEs), respectively, as shown.

Recently, several studies used high-throughput mRNA sequencing (RNA-seq) to study splicing throughout normal hematopoiesis (Fig. 1A). These include RNA-seq of normal human hematopoietic stem cells (HSCs) plus seven other progenitor populations (Chen et al. 2014b), terminal human (Pimentel et al. 2016) and murine erythropoiesis (Edwards et al. 2016), terminal murine granulopoiesis (Wong et al. 2013), and terminal murine megakaryopoiesis (Edwards et al. 2016). One overlapping concept illustrated by these studies is that each stage of hematopoiesis is defined by cell type-specific differential splicing, which may not be associated with appreciable changes in parental gene expression. In this model, specific splicing events occur at defined stages of hematopoiesis, resulting in selective increases or decreases in inclusion of individual exons to alter the function and/or stability of the encoded proteins to define a cell's identity. Further studies of this model are needed, however, as only a small number of cell type-specific splicing events have been functionally defined in normal hematopoiesis (one of the best-characterized examples is shown in Fig. 1B).

Stage-specific regulation of splicing has been well characterized in the development of other tissue types, such as neuronal differentiation (for recent reviews, see Licatalosi and Darnell 2010; Zheng and Black 2013) and heart development (van den Hoogenhof et al. 2016). As just one example, the development of post-mitotic neurons from neuronal precursor cells (NPCs) is regulated in part by a change in the expression levels of two splicing regulatory proteins: the polypyrimidine (Py) tract-binding proteins 1 and 2 (PTBP1 and PTBP2, also known as PTB and nPTB). In NPCs, PTBP1 levels are elevated and repress PTBP2 levels by promoting expression of an isoform of *PTBP2* that undergoes nonsense-mediated decay (NMD). However, as NPCs differentiate, PTBP1 is silenced, resulting in up-regulation of PTBP2 and subsequent promotion of a neural AS program (Boutz et al. 2007; Keppetipola et al. 2012). Whether analogous splicing regulatory mechanisms similarly regulate hematopoietic differentiation remains an open question.

The concept of differentiation stage-specific switches in RNA processing with corresponding effects on protein structure has been most clearly defined within hematopoiesis from studies of the terminal stages of granulopoiesis (Wong et al. 2013) and erythropoiesis (Edwards et al. 2016; Pimentel et al. 2016), stages of blood development associated with distinct morphological changes and changes in the proteome (including cell surface protein expression). RNA-seq analysis of promyelocytes, myelocytes, and mature granulocytes isolated from murine bone marrow (BM) revealed a striking pattern of intron retention (IR) that increased during granulopoiesis (Wong et al. 2013). Many intron-containing mRNAs were apparently efficiently exported from the nucleus and subject to degradation by NMD due to premature termination codons (PTCs) contained within the retained introns. For example, accumulation of IR transcripts was observed in *Lmnb1*, which encodes a key nuclear lamin protein, resulting in down-regulation of Lmnb1. Enforced expression of intronless *Lmnb1* resulted in aberrant granulocytes with altered nuclear structure and volume. Increased IR in the final stages of granulopoiesis was coincident with decreased levels of mRNAs encoding several core spliceosomal proteins, including members of the U1 and U2 small nuclear ribonucleoprotein (snRNP) complexes, potentially contributing to inefficient intron excision (whether snRNA levels are similarly lower remains to be determined).

Analogous to the above studies of granulopoiesis, RNA-seq analysis of five populations of human erythroid precursors representing the last four cell divisions before enucleation similarly revealed striking increases in levels of intron-containing transcripts in the final two stages of erythropoiesis (Pimentel et al. 2016). In contrast to the report by Wong et al. (2013) that many intron-containing transcripts were apparently exported to the cytoplasm during granulopoiesis, Pimentel et al. (2016) found that many intron-containing transcripts were retained in the nucleus during erythropoiesis. Because these transcripts were retained in the nucleus, they were not degraded by NMD, which is translation-dependent and occurs in the cytoplasm. However, in either case, IR resulted in depressed levels of the encoded full-length protein. A more recent analysis of murine erythropoiesis and megakaryopoiesis similarly revealed alterations in IR during the development of both of these populations as well (Edwards et al. 2016). These studies suggest that IR is a common mechanism used to couple alterations in splicing and gene expression during hematopoietic differentiation. Given that intron-containing transcripts may be subjected to impaired nuclear export as well as cytoplasmic RNA degradation, further efforts to characterize nuclear versus cytoplasmic RNAs during hematopoietic differentiation may identify novel additional features of RNA processing during hematopoiesis. Finally, while increased IR appears to inversely correlate with gene expression in both terminal erythropoiesis (Edwards et al. 2016) and granulopoiesis (Wong et al. 2013), it is possible that a proportion of IR-retained transcripts undergoes translation to generate novel proteins. Use of techniques such as ribosome profiling of purified hematopoietic populations may help to identify unexpected translation of such intron-containing transcripts.

Forms of splicing regulation in addition to IR likely also generate PTCs to regulate gene expression during normal cellular differentiation. As one example, every member of the human serine/arginine-rich (SR) gene family has a PTC-containing isoform that is targeted for degradation by NMD (Lareau et al. 2007; Ni et al. 2007). Indeed, mRNAs encoding several SR proteins are down-regulated during terminal human erythropoiesis, concordant with higher expression of their PTC-containing isoform counterparts (Pimentel et al. 2016). Thus, further efforts to comprehensively characterize changes in splicing beyond IR during hematopoietic differentiation are still needed. In addition, efforts are needed to identify *trans*-acting splicing regulatory proteins that contribute to stage-specific switches in hematopoietic differentiation (as described above for PTBP1/2 regulation of neuronal development).

The above studies illustrate how global alterations in splicing catalysis can couple splicing to the regulation of total protein produced from particular genes. This connection complements AS's better-known role in regulating the production of specific protein isoforms and indicates how splicing provides an additional mechanism of gene expression regulation in addition to better-studied modes of transcriptional, translational, and post-translational regulation of gene expression in normal hematopoiesis.

In addition to identifying specific splicing events that help define different stages of hematopoiesis, these recent RNA-seq studies also highlighted important deficiencies in our knowledge of normal splicing. For example, the Blueprint consortium's study of splicing in normal human hematopoietic stem and progenitor cells (HSPCs) (Chen et al. 2014b) reported that 26.5% of putative splice junctions identified in their data set were previously unannotated [not found in the Ensemble version 70, University of California at Santa Cruz (UCSC) expressed sequence tag (EST)/mRNA data set or the poly(A)^+^ RNA-seq data set from Illumina]. Pimentel et al. (2016) similarly reported specific isoforms in proerythroblasts that were not present in the RefSeq transcript set. Thus, further efforts to map the specific isoforms that are present in different stages of normal human hematopoiesis will almost certainly be needed to define how those events are altered in MDSs and other clonal disorders of hematopoiesis.

## Discovery of spliceosomal gene mutations and their molecular consequences

**Figure 2.**
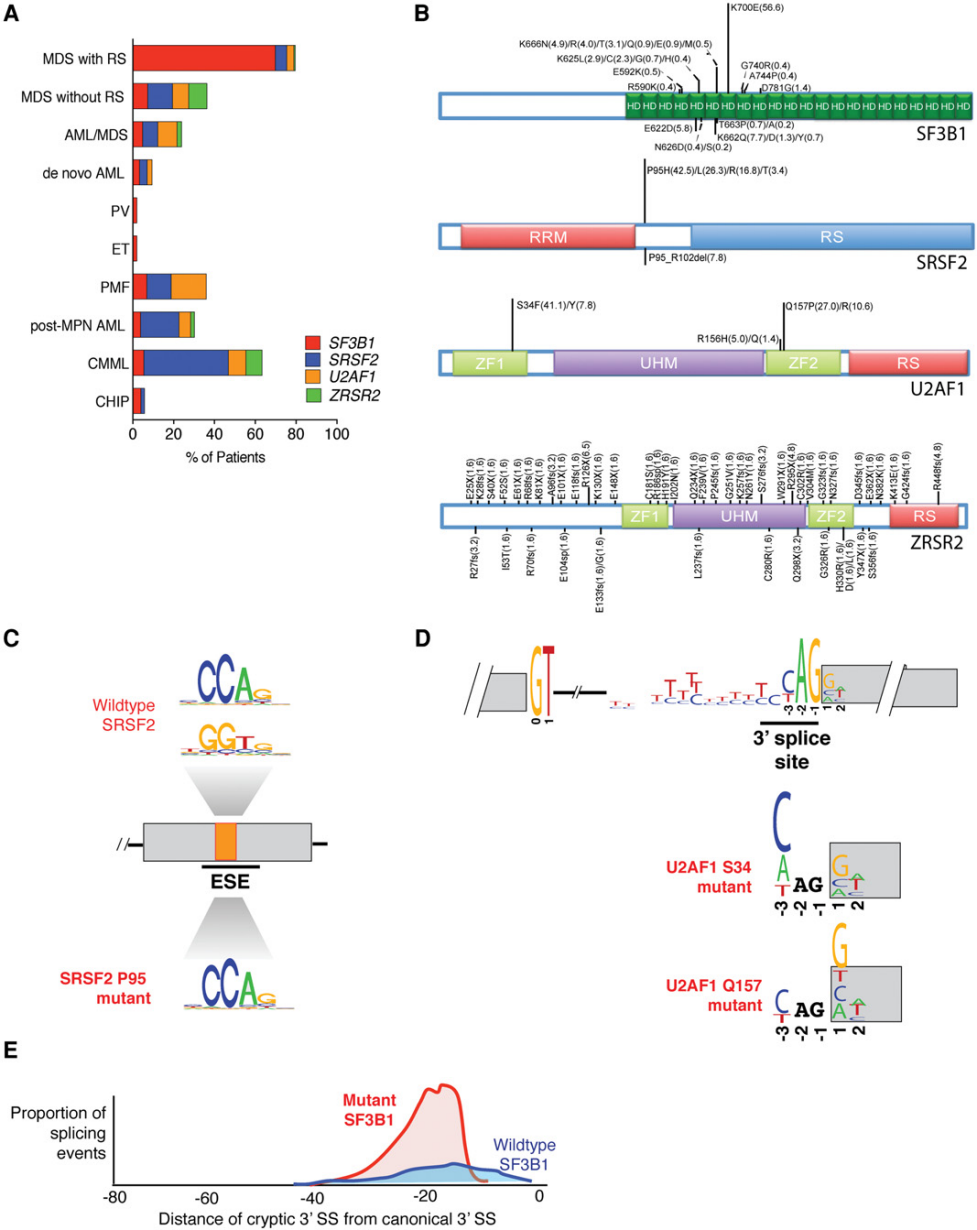
Genetic and bioinformatic characterization of spliceosomal gene mutations and their effects on splicing in myeloid malignancies. (*A*,*B*) Frequencies of spliceosomal gene mutations (*A*) and proportion of mutated residues across myeloid malignancies (*B*) (references used to make this figure are noted in the Supplemental Material). Known domains of each mutated protein are shown in colored boxes. The height of each bar is proportional to the frequency of mutations affecting each residue. The frequency of mutations represented by each amino acid change is indicated in the parentheses. (HD) HEAT domain repeat; (RRM) RNA recognition motif; (RS) arginine–serine rich domain; (ZF) zinc finger; (UHM) U2AF homology domain. (*C*–*E*) Current understanding of the mechanistic effects of mutations in *SRSF2* (*C*), *U2AF1* (*D*), and *SF3B1* (*E*) on RNA splicing. As shown in *C*, *SRSF2* mutations affecting Pro95 alter the recognition of exons containing specific ESE sequences. Wild-type SRSF2 normally binds to and promotes splicing of C- and G-rich ESEs equally well, while mutant SRSF2 has skewed preference for C-rich ESEs (Kim et al. 2015; Zhang et al. 2015). (*D*) In contrast, *U2AF1* mutations promote or repress splice site (SS) recognition based on the identity of the nucleotide at the −3 or +1 sites that flank the AG of the 3′ SS. This occurs in an allele-specific manner in which *U2AF1* S34 mutations drive alterations at the −3 position (with a C/A>>T preference), and mutations at Q157 drive alterations at the +1 position (with a G>>A preference) (Ilagan et al. 2015; Okeyo-Owuor et al. 2015; Shirai et al. 2015). Finally, *SF3B1* mutations have been shown in three studies to promote use of 3′ cryptic SSs that are 10–30 base pairs (bp) upstream of the canonical 3′ SS (Darman et al. 2015; DeBoever et al. 2015; Alsafadi et al. 2016).

As mentioned earlier, in late 2011, frequent mutations in genes encoding spliceosomal proteins were discovered and subsequently shown to be the most common category of mutations in MDSs, related myeloid malignancies, and clonal disorders of hematopoiesis that do not meet the criteria for malignancy (so-called “clonal disorders of hematopoiesis of indeterminate potential” [CHIP]) (Fig. 2A; Papaemmanuil et al. 2011, 2013; Yoshida et al. 2011; Graubert et al. 2012; Haferlach et al. 2014; Jaiswal et al. 2014; Genovese et al. 2015). The vast majority of these mutations is concentrated in one of three genes: *SF3B1*, *U2AF1*, or *SRSF2*. Interestingly, the mutations occur entirely as heterozygous missense mutations at highly restricted residues and also occur in a mutually exclusive manner with one another (Fig. 2B). These genetic features, along with the absence of recurrent nonsense or frameshift mutations in these three genes, suggest that the mutations may confer an alteration of function, such as a neomorphic and/or dominant-negative activity. Given that the roles of these proteins are most established in the context of RNA splicing, a number of studies have recently been performed to evaluate the mechanistic effects of these mutations on RNA splicing. The general findings from these studies are described and appraised below.

### Mutations in SRSF2 alter RNA splicing in a sequence-specific manner and are distinct from loss-of-function mutations

Two recent studies provided independent and consistent data showing that mutations in *SRSF2* alter the protein's role in RNA splicing in a manner completely distinct from loss of function (Kim et al. 2015; Zhang et al. 2015). Although not a member of the core spliceosome, SRSF2 is a member of the SR protein family that facilitates spliceosome assembly by binding to exonic splicing enhancer (ESE) sequences within pre-mRNAs through its RNA recognition motif (RRM) domain (Graveley and Maniatis 1998; Liu et al. 2000). SRSF2 normally recognizes ESE elements within the pre-mRNA with a consensus motif of SSNG (where S represents C or G) to promote exon recognition (Daubner et al. 2012). In the wild-type state, SRSF2 binds ESEs with the motif sequence of CCNG and GGNG equally well to promote inclusion of the exons bearing these motifs.

Mutations in *SRSF2* consistently affect the proline 95 (Pro95) residue and occur as heterozygous missense mutations or, rarely, in-frame insertion or deletion mutations that also affect the Pro95 residue. Unbiased genome-wide analysis of RNA-seq data from isogenic genetically modified murine hematopoietic cells as well as K562 cells bearing a substitution of histidine for Pro95 (P95H) in SRSF2 revealed that mutant SRSF2's ability to promote splicing varied based on the exonic SSNG motif. More specifically, cells expressing mutant SRSF2 exhibit enrichment for CCNG ESE motifs in cassette exons with increased inclusion, whereas GGNG ESE motifs are enriched in repressed cassette exons (Fig. 2C). Notably, the sequence-specific requirement of mutant SRSF2 to promote or repress splicing was verified by mutagenizing the ESE motifs in a series of minigene experiments modeling mutant SRSF2-responsive splicing events in both studies (Kim et al. 2015; Zhang et al. 2015). As further evidence of the sequence-specific effects of mutant SRSF2 on splicing, overexpression of wild-type SRSF2 in hematopoietic cells was associated with increased inclusion of cassette exons bearing either CCNG or GGNG ESE motifs. In contrast, depletion of SRSF2 was associated with decreased inclusion of cassette exons bearing either ESE motif. Moreover, when the spatial distribution of CCNG and GGNG motifs across genomic loci containing cassette exons promoted or repressed in association with *SRSF2* mutations was examined, these motifs were found enriched specifically over cassette exons and not over the flanking introns or exons (Kim et al. 2015). This spatially restricted enrichment of specific ESEs in association with mutant SRSF2-driven splicing changes further reinforced the concept that SRSF2 mutations alter SRSF2's normal ESE recognition activity.

### Mutations in SRSF2 alter RNA-binding preferences, consistent with altered exon recognition

The above data establishing that *SRSF2* mutations change the protein's normal ESE preferences led to the question of whether this was due to direct alterations in RNA-binding affinity created by Pro95 mutations. Here again, distinct methods used by two independent groups led to a similar conclusion: The enrichment/depletion of specific ESEs in cassette exons in the presence of *SRSF2*P95H mutations is due to altered SRSF2:RNA interactions (Kim et al. 2015; Zhang et al. 2015). Isothermal calorimetry and RNA gel shift assays of the interactions between wild-type or mutant SRSF2 peptide (amino acids 1–101, consisting of the RRM and linker domain of SRSF2) and RNA ligands bearing various SSNG sequences revealed that mutant SRSF2 binds RNA oligonucleotides with C-rich ESEs threefold to fourfold more avidly than wild-type SRSF2. In contrast, mutant SRSF2 exhibited a 1.2-fold to 2.1-fold decrease in binding affinity to RNA oligonucleotides with G-rich ESE sequences.

Both direct RNA-binding affinity measurements and informatic computation of ESE motif preferences revealed the same hierarchy of motif preferences for mutant SRSF2 (CC>GC>CG>GG). However, exactly how Pro95 mutations confer this change in RNA-binding preference is still not clear. Although the Pro95 residue is not part of the canonical RRM, recent nuclear magnetic resonance (NMR) data suggest that the Pro95 residue plays a role in SRSF2's RNA-binding activity. Interestingly, NMR titration experiments using the RRM domain of wild-type and mutant forms of SRSF2 with RNA oligos suggested that mutations at Pro95 alter the conformation of the termini of the RRM without affecting the RNA-binding surface of the SRSF2 RRM (Kim et al. 2015). Future efforts to resolve the structure of SRSF2 will be important to elucidate the structural effects of Pro95 mutations on interactions with RNA. In addition, although both studies described above indicate that *SRSF2* mutations affect its direct interactions with RNA, these mutations might also directly or indirectly alter SRSF2 interactions with other spliceosomal proteins. *SRSF2* mutations reportedly do not affect interactions between SRSF2 and several select spliceosomal proteins (SF3B1, U2AF1, or snRNP70) (Zhang et al. 2015), but further work to understand the potential effects of *SRSF2* mutations on a broader network of interacting proteins is needed.

### Mutations in U2AF1 alter RNA splicing in a sequence-specific manner and are allele-specific

The earliest attempts to understand the functional implications of spliceosomal mutations on RNA splicing focused on *U2AF1* (Yoshida et al. 2011). U2AF1 is the small subunit of the U2AF heterodimer that normally recognizes the consensus motif yAG|r (y = pyrimidine and r = purine) at the intron|exon boundary (Merendino et al. 1999; Wu et al. 1999; Zorio and Blumenthal 1999; for a very recent review of the role of the U2AF heterodimer in splicing, see Wu and Fu 2015). More than 10 distinct mutations have been identified in *U2AF1*, with nearly all occurring at residues S34 or Q157, each of which is present in one of the zinc finger domains of U2AF1 (Fig. 2B).

Several studies using unbiased genomic analysis of isogenic human and murine cells expressing ectopic mutant or wild-type U2AF1 have now revealed that mutations in *U2AF1* consistently alter 3′ splice site (SS) preference in a sequence-specific manner based on the identity of the nucleotide surrounding the AG dinucleotide that forms the 3′ SS's core consensus motif (Przychodzen et al. 2013; Brooks et al. 2014; Ilagan et al. 2015; Okeyo-Owuor et al. 2015; Shirai et al. 2015). Specifically, expression of *U2AF1*S34F/Y promotes recognition of the 3′ SS bearing a C or A immediately preceding the AG and represses those bearing a T at this position (Fig. 2D). As with SRSF2, this sequence-specific alteration driven by mutant U2AF1 is distinct from splicing alterations that occur with U2AF1 depletion, indicating that *U2AF1* mutations cause alteration, not loss, of function (Ilagan et al. 2015). Interestingly, analysis of the effects of Q157P/R mutations revealed that these mutations also affected 3′ SS recognition but in a distinct manner. Q157P/R mutations promoted recognition of the 3′ SS bearing a G immediately following (rather than preceding) the AG and repressed those bearing an A at this position (Fig. 2D; Ilagan et al. 2015). As with SRSF2, the exact structural basis for how disease-associated mutations in *U2AF1* alter RNA-binding affinity needs further clarification.

Many factors are involved in correct SS recognition in complex with U2AF1, including U2AF2, hnRNPA1, and DEK, among others. Thus, future efforts are needed to determine whether S34 and Q157 mutations in *U2AF1* alter the extensive protein–protein interaction networks across the exon and the intron that are necessary for spliceosome assembly.

### SF3B1 hot spot mutations appear to promote use of cryptic 3′ SSs

Despite being the most common mutations affecting the spliceosome in MDSs, the mechanistic consequences of mutations in *SF3B1* have been less clear than those of *SRSF2* or *U2AF1*. SF3B1 is a member of the U2 snRNP complex, which is responsible for 3′ SS recognition. The U2 snRNP is directed to the 3′ SS by short, conserved pre-mRNA sequences, including the branch point sequence (BPS; a degenerate sequence motif usually located 21–34 base pairs [bp]) upstream of the 3′ SS), the Py tract, and the AG dinucleotide at the intron–exon junction (described earlier) (for a recent review of the basic mechanisms of normal splicing, see Wahl and Luhrmann 2015). The mutations in *SF3B1* largely cluster within the C-terminal HEAT domains (residues 622–781), with the most commonly mutated residue being K700.

Three recent studies have reported that cells with mutations in *SF3B1* are associated with alterations in splicing specifically due to aberrant 3′ SS selection (Fig. 2E; Darman et al. 2015; DeBoever et al. 2015; Alsafadi et al. 2016). In each of these reports, SF3B1 mutant cells were found to use cryptic AG dinucleotides lying 10–30 bp upstream of the canonical AG dinucleotide. Although this alteration in splicing was not assayed in myeloid hematopoietic cells, it was seen in patient transcriptomes from a variety of other histologies as well as isogenic Nalm-6 cells (a pre-B lymphoblastic leukemia cell line) bearing SF3B1 mutations in their endogenous locus.

In the study by Darman et al. (2015), the motif sequences associated with cryptic and canonical AGs in mutant SF3B1-expressing cells were studied and revealed an enrichment of pyrimidines upstream of the canonical AG, while the cryptic AGs were associated with a short and weak Py tract and an enrichment of adenines. Darman et al. (2015) generated a minigene of a splicing event that was consistently altered in association with all studied hot spot mutations in *SF3B1* (exon 9–exon 10 junction in *ZDHHC16*) and used a minigene mutagenesis approach to study the contributions of the cryptic 3′ SS, the length/strength of the Py tract, and the potential BPS to splicing in the presence of wild-type versus mutant SF3B1. A series of elegant experiments using this and similar minigenes revealed that (1) the cryptic AG is necessary to induce aberrant splicing by mutant SF3B1, (2) the cryptic AG requires the downstream canonical Py tract for recognition, and (3) distinct BPSs are used in cells expressing wild-type versus mutant SF3B1 (Darman et al. 2015). However, it remains unknown whether these specific mechanisms generalize to the broader spectrum of cryptic 3′ SSs that are activated by *SF3B1* mutations.

Normally, the binding of U2 snRNP and other spliceosomal proteins around the BPS prevents 3′ SS selection in a 12- to 18-bp region directly downstream from the BPS due to steric hindrance (Fig. 2E; Smith et al. 1993; Chua and Reed 2001). Informatic characterization of the sequence features around the cryptic 3′ SSs seen by DeBoever et al. (2015) in SF3B1 mutant primary patient transcriptomes indicated that many cryptic 3′ SS used in SF3B1 mutant cells fell within this normally sterically protected region downstream from the BPS. DeBoever et al. (2015) therefore proposed that *SF3B1* mutations relieve the steric occlusion of this region, thereby permitting recognition of normally obscured cryptic 3′ SSs.

The study by Darman et al. (2015) highlighted that the majority of cryptic AGs generated in SF3B1 mutant cells were located upstream of the canonical 3′ SSs and induced frameshifts, potentially causing many of the resulting transcripts to be degraded by NMD (Darman et al. 2015). Specific cryptic 3′ SSs in the region downstream from the BPS play roles in some inherited diseases (including those due to disrupted tumor suppressor genes such as *ATM*, *NF1*, and *TP53*) (Kralovicova et al. 2005). However, widespread usage of cryptic 3′ SSs due to a cancer-associated mutation has not been previously described. This enrichment in cryptic 3′ SSs associated with mutant SF3B1 is also distinct from altered splicing in cancer due to somatic mutations at SSs (which appear to most commonly affect 5′ SS recognition) (Jung et al. 2015). Despite this recent progress, however, our understanding of how mutations in SF3B1 result in altered 3′ SS usage is relatively incomplete. Progress is limited by the current lack of understanding of the normal function of SF3B1's HEAT domains, where the majority of *SF3B1* mutations are located.

### Global splicing changes in spliceosome mutant cells are subtle

Despite the observations made so far regarding global splicing effects of spliceosomal mutations, it is important to note that only a small proportion of splicing events is affected by these mutations. For instance, cryptic 3′ SSs are used in only a small fraction of genes in *SF3B1* mutant cells, and they are used relatively infrequently even in that subset of genes (<10% relative to the canonical 3′ SSs for most affected genes) (Darman et al. 2015; DeBoever et al. 2015). Similarly modest global effects were observed for *U2AF1* and *SRSF2* mutations. Fewer than ∼5% of alternatively spliced events exhibited changes in isoform usage of >10% in isogenic cells bearing these mutations, and most changes in isoform usage were relatively modest (Ilagan et al. 2015; Kim et al. 2015; Zhang et al. 2015). Nonetheless, these observations do not eliminate the potential for functionally important effects of specific individual splicing events and their attendant downstream sequelae on hematopoiesis.

It remains possible that spliceosomal mutations induce more dramatic global splicing changes in specific contexts. Initial efforts to understand the genome-wide effects of the *U2AF1*S34F mutation reported widespread production of abnormal mRNAs that are substrates for degradation by NMD in association with mutant U2AF1 overexpression (Yoshida et al. 2011). However, this observation was not recapitulated in cells with more modest ectopic overexpression of U2AF1 or in the transcriptomes of patients with *U2AF1* mutations (Brooks et al. 2014; Ilagan et al. 2015; Shirai et al. 2015).

### Mutations in ZRSR2 appear to be consistent with loss of function and impair splicing of U12-type introns

As noted above, the majority of mutations affecting spliceosomal proteins occurs in *SF3B1*, *SRSF2*, and *U2AF1*. However, the discovery of these mutations by Yoshida et al. (2011) also identified recurrent mutations in *ZRSR2* across the entire length of the gene. Yoshida et al. (2011) observed nonsense, frameshift, SS, and missense mutations (Fig. 2B), in stark contrast to the hot spot missense mutations in *SF3B1*, *SRSF2*, and *U2AF1*. Moreover, mutations in *ZRSR2*, which is encoded in Xp22.1, occur most commonly in males. These data strongly suggest that the mutations cause loss of function.

ZRSR2 is a component of the U12 snRNP and contacts the 3′ SSs of U12-type introns (Shen et al. 2010). U12-type introns are a rare class of introns that are removed by a dedicated, U12-dependent “minor” spliceosome that is distinct from the U2-dependent “major” spliceosome (Hall and Padgett 1994; Tarn et al. 1995; Tarn and Steitz 1996; for a recent review, see Niemela and Frilander 2014). U12-type introns comprise only ∼0.5% of all human introns (Sheth et al. 2006; Alioto 2007). Intriguingly, a subset of U12-type introns is spliced relatively inefficiently relative to their U2-type counterparts. This inefficient splicing can result in nuclear retention of mRNAs containing unspliced U12-type introns, possibly followed by nuclear decay, resulting in a regulatory mechanism analogous to that observed by Pimentel et al. (2016) in erythropoiesis.

Thus far, only one report has studied the mechanistic implications of ZRSR2 loss on splicing in hematopoiesis. Madan et al. (2015) used TF-1 cells with RNAi-mediated down-regulation of *ZRSR2* as well as primary MDS patient transcriptomes that were wild type or mutant for *ZRSR2.* RNA-seq analysis of these cells revealed that IR was enriched in *ZRSR2* mutant or knockdown samples relative to *ZRSR2* wild-type cells as well as normal BM cells. Categorization of the introns affected as U2- or U12-type revealed that U12-type introns were preferentially affected by ZRSR2 loss. Although prior in vitro splicing assays suggested that ZRSR2 contributes to efficient splicing of both U2- and U12-type introns (Shen et al. 2010), Madan et al. (2015) observed that effects on U2-type introns were mostly restricted to transcripts where U12-type introns were also affected (most genes that depend on the minor spliceosome for processing contain a single U12-type intron surrounded by U2-type introns [Alioto 2007; Chang et al. 2007]). Gene ontology (GO) analysis of genes that were misspliced in association with *ZRSR2* mutation or loss revealed enrichment for genes involved in MAPK and ErbB signaling, including predicted IR affecting genes encoding key cytokine signaling intermediates such as PTEN, BRAF, ARAF, and RAF1 as well as E2F transcription factors (Madan et al. 2015). These are intriguing findings that need to be validated at the protein level and functionally linked to MDS disease pathogenesis. Possibly consistent with *ZRSR2* mutations predominantly affecting minor intron splicing is the fact that *ZRSR2* mutations may coexist with mutations in *SF3B1*, *SRSF2*, and/or *U2AF1* (which presumably primarily impact splicing of major introns), although this putative co-occurrence needs to be validated using clonal assays (Yoshida et al. 2011).

## Biological implications of spliceosome alterations on hematopoiesis

**Figure 3.**
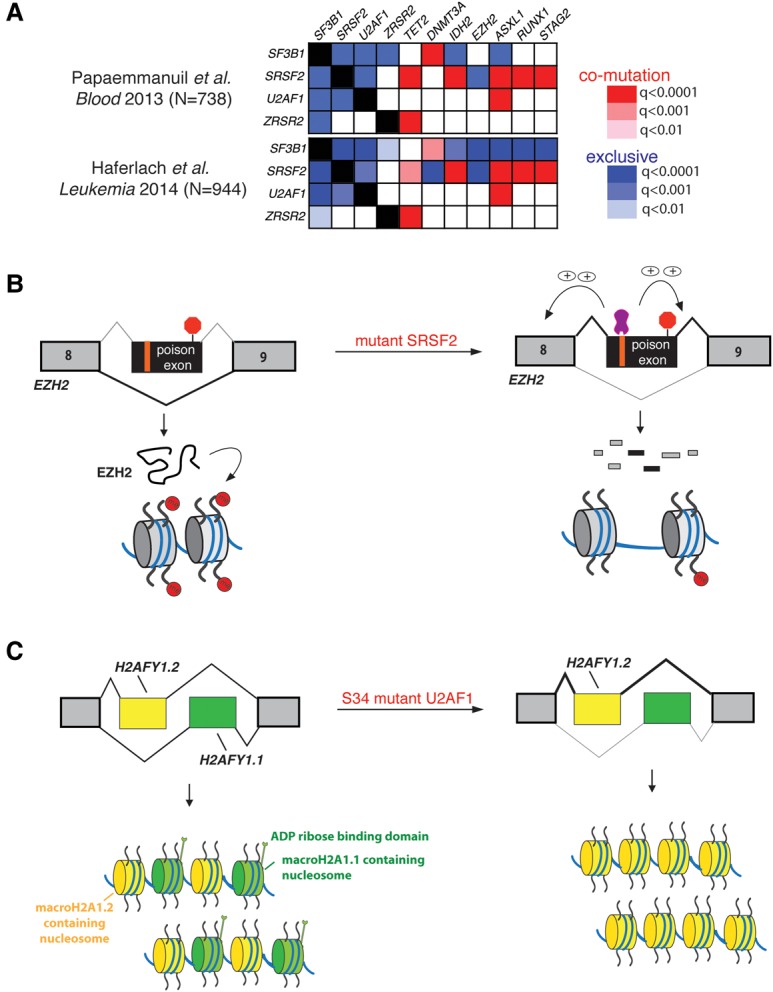
Mechanisms linking spliceosomal gene mutations to myeloid malignancy pathogenesis. (*A*) The pattern of genetic alterations significantly coexisting with, or mutually exclusive of, individual spliceosomal gene mutations. The *q*-values testing mutational co-occurrences from two separate MDS sequencing studies involving a total of >1600 MDS patients are shown (Papaemmanuil et al. 2013; Haferlach et al. 2014). The degree of red and blue shading corresponds to the *q*-value for mutational co-occurrence and exclusivity, respectively, between mutations in each spliceosomal gene and the other genes listed. (*B*) The effect of SRSF2 mutations on EZH2 expression and consequent histone H3 Lys27 trimethylation. Mutant *SRSF2* promotes inclusion of a cassette exon that results in an isoform of *EZH2* that undergoes NMD. This event has been validated at the mRNA and protein level, and a mutant *SRSF2*-responsive ESE that controls the *EZH2* splicing change has been identified (Kim et al. 2015). (*C*) The effects of *U2AF1*S34 mutations on *H2AFY* splicing. Although this splicing event has been validated in several reports, the downstream effects on macroH2A1 isoform expression and distribution and consequent biological effects of altered macroH2A1.1/macroH2A1.2 ratios have not been studied in the context of hematopoiesis.

While the observation that mutations in spliceosomal genes present across MDS patients are mutually exclusive might suggest similar effects of each of these mutations on MDS pathogenesis, several facts hint that these mutations might not be simply converging on common downstream effects. First, as described above, it appears that different spliceosomal gene mutations have distinct mechanistic effects on splicing. Moreover, clinical correlative studies have revealed a strong relationship between specific spliceosomal gene mutations and different clinical phenotypes among myeloid malignancies (Fig. 2A). For example, mutations in *SF3B1* are highly enriched in MDS patients with ringed sideroblasts (Papaemmanuil et al. 2011; Yoshida et al. 2011), which represents an indolent form of MDS marked by hyperplasia of erythroid precursor cells with aberrant accumulation of mitochondrial ferritin around the nucleus. In contrast, mutations in *SRSF2* are highly enriched in the MDS/myeloproliferative syndrome chronic myelomonocytic leukemia (CMML) (Yoshida et al. 2011; Meggendorfer et al. 2012). In addition to these unique phenotypic correlations, mutations in spliceosomal factors are also each associated with particular sets of coexisting and mutually exclusive genetic alterations, again consistent with different pathogenic mechanisms unique to each mutation (Fig. 3A).

In contrast to the advances made so far in defining the mechanistic implications of spliceosomal mutations for RNA splicing, identifying specific misspliced events that are functionally linked to MDS disease pathogenesis is in its earliest stages. Below, we review published and ongoing efforts to understand how the global effects of each mutated splicing factor on RNA splicing may promote the development of specific subtypes of myeloid malignancies.

### SF3B1 mutations and heme synthesis

One of the most interesting findings resulting from the discovery of spliceosomal gene mutations was an unexpected and highly significant association between *SF3B1* mutations and MDS subtypes with ringed sideroblasts (Malcovati et al. 2015). Somatic *SF3B1* mutations are present in >90% of refractory anemia with ringed sideroblasts (RARS) MDS patients as well as ∼70% of patients with non-RARS forms of MDS with RSs, such as refractory cytopenia with multilineage dysplasias and ringed sideroblasts (RCMD-RS) and RARS associated with marked thrombocytosis (RARS-T).

Ringed sideroblasts are a characteristic feature of a group of disorders known as sideroblastic anemias (SAs), which include congenital, nonclonal disorders as well as acquired clonal disorders such as RARS MDSs. Mutations affecting various stages of iron metabolism, uptake, and trafficking have been found in patients with congenital SA. These include mutations in *ALAS2* (5′-aminolevulinate synthase 2) seven-codon in X-linked SA (Cotter et al. 1992), mutations in *ABCB7* in X-linked SA with spinocerebellar ataxia (Allikmets et al. 1999), and mutations in *GLRX5* (glutaredoxin 5) in congenital SA (Camaschella et al. 2007; Ye et al. 2010), among many others (for a recent review, see Cazzola and Malcovati 2015). Although altered expression of some of these genes, including down-regulation of *ABCB7* and up-regulation of *ALAS2*, had been known to be present in RARS (Boultwood et al. 2008; Nikpour et al. 2010) for some time, mutations in the genes found in congenital SA have not been described in MDSs.

RNA-seq analyses of RARS samples directly from BM or in vitro primary erythroid cultures suggested that aberrant splicing of *SLC25A37* (Mitoferrin-1), resulting in an IR event (Visconte et al. 2015), and/or *ABCB7*, resulting in an NMD substrate, is associated with the presence of *SF3B1* mutations (Darman et al. 2015). The putative *ABCB7* missplicing event (characterized as use of a cryptic 3′ SS between exons 8 and 9 in *ABCB7*, causing the addition of a PTC in a seven-codon addition to the protein sequence prior to the start of exon 9) (Darman et al. 2015) is particularly intriguing, as forced down-regulation of ABCB7 in normal CD34^+^ cells is associated with impaired in vitro erythroid differentiation (Nikpour et al. 2013). Moreover, restoration of *ABCB7* levels by cDNA overexpression in CD34^+^ BM cells from RARS patients appears to functionally rescue impaired in vitro erythroid colony output (although the morphological effect of ABCB7 modulation on ringed sideroblasts themselves was not evaluated in those assays) (Nikpour et al. 2013). However, rigorous confirmation of the consequences of missplicing of *ABCB7* and *SLC25A37* for protein levels in the proper cell context is needed, as is mechanistic determination of how mutant SF3B1 induces missplicing of these potential target genes. Moreover, despite these potential links between mutant SF3B1 and aberrant heme synthesis, putative targets linking aberrant splicing mediated by *SF3B1* mutations and the clonal hematopoiesis that characterizes RARS have not been defined. One early report suggested that constitutive heterozygous *Sf3b1* knockout mice had marrow ringed sideroblasts (Visconte et al. 2012), but this feature was not confirmed in two later independent reports using the same murine model (Matsunawa et al. 2014; Wang et al. 2014). It is also important to note that even *Abcb7* conditional knockout mice do not exhibit ringed sideroblasts (Pondarre et al. 2007), potentially highlighting a currently unknown difference between human and murine erythroid precursors with respect to mitochondrial iron metabolism.

### Effects of SRSF2 and U2AF1 mutations on epigenetic regulatory proteins

In contrast to the association of *SF3B1* mutations with ringed sideroblast-containing subtypes of MDS, *SRSF2* and *U2AF1* mutations appear to be enriched in more aggressive subtypes of MDS, including the WHO subtypes refractory anemia with excess blasts I (RAEB I) and RAEB II. While spliceosomal gene mutations were thought to be rare in de novo acute myeloid leukemia (AML) based on initial reported frequencies of 6.6% by Yoshida et al. (2011) and <5% in The Cancer Genome Atlas (The Cancer Genome Atlas Research Network 2013)*,* subsequent analyses of elderly AML (>60-yr-old) cohorts revealed spliceosomal mutations in 20%–40% of such patients, the majority of which is *SRSF2* mutations (Silva et al. 2015).

As is the case for *SF3B1* mutations*,* efforts to link *SRSF2* and *U2AF1* gene mutations with aberrant HSC self-renewal and myeloid malignancy are still nascent, although one concrete link has been established for *SRSF2*. Mutations in *SRSF2* were found to promote a specific PTC-containing isoform of *EZH2*, and minigene assays were subsequently used to identify the specific ESE that was responsible for altered *EZH2* splicing in the presence of mutant SRSF2 (Fig. 3B; Kim et al. 2015)*.* The PTC-containing isoform of *EZH2* was degraded by NMD, resulting in decreased levels of EZH2 protein itself as well as the histone H3 Lys27 trimethylation (H3K27me3) mark that it catalyzes. Finally, restoration of normally spliced EZH2 expression by cDNA rescue partially rescued the hematopoietic defects of *Srsf2* mutant HSPCs. These data clearly linked mutations in *SRSF2* to aberrant epigenetic regulation through insufficient EZH2 expression and likely underlie the known but unexplained mutual exclusivity between loss-of-function mutations in *EZH2* and *SRSF2* mutations (Fig. 3A; Papaemmanuil et al. 2013; Haferlach et al. 2014). Further efforts are now needed to define the functional consequences of other splicing events altered by mutant SRSF2 that were identified by Kim et al. (2015) and Zhang et al. (2015).

A number of interesting and potentially functionally relevant misspliced events have also been described in the setting of mutant U2AF1. As with SRSF2-driven missplicing, several of these events also result in altered splicing of epigenetic regulatory genes, some of which are recurrently mutated in myeloid malignancies and/or associated with clonal hematopoiesis. These include altered splicing of *BCOR*, *ASXL1*, and *DNTM3B* (Ilagan et al. 2015; Shirai et al. 2015). The functional consequences of each splicing change needs to be determined in detail, especially given the known statistical enrichment of mutations in *ASXL1* and *U2AF1* in myeloid malignancies (Fig. 3A; Papaemmanuil et al. 2013; Haferlach et al. 2014).

Perhaps one of the most intriguing potential links between splicing mutations and altered epigenetic regulation is skewed splicing of *H2AFY* (Ilagan et al. 2015; Shirai et al. 2015), which encodes the histone variant macroH2A1, in association with mutant U2AF1. *H2AFY* contains two mutually exclusive exons, generating the two isoforms macroH2A1.1 and macroH2A1.2 of macroH2A1. The functions of these two isoforms differ, with important functional and prognostic implications in other forms of cancer (Fig. 3C; Novikov et al. 2011; Dardenne et al. 2012; Chen et al. 2014a). Although these two macroH2A1 isoforms have not been well studied in the context of hematopoiesis, from the study of other tissue types, it appears that the two macroH2A1 isoforms differ in their genome-wide distributions and ability to undergo post-translational modifications to influence gene expression. MacroH2A1.1 tends to localize to transcriptionally active acetylated euchromatin, while macroH2A1.2 tends to be associated with heterochromatin marked by H3K27me3 (Chen et al. 2014a). This association is thought to occur due to the fact that macroH2A1.1 binds ADP-ribose-related molecules, including poly(ADP-ribose) (PAR) (Kustatscher et al. 2005). PAR in turn associates with the histone acetyltransferase CBP, which promotes acetylation of chromatin in the region surrounding macroH2A1.1 (Chen et al. 2014a). In contrast, macroH2A1.2 lacks the ability to bind PAR. Further analysis of the role of *H2AFY* splicing and macroH2A1 isoforms in malignant hematopoiesis is just one of the many potentially important avenues of investigation highlighted by analyses of target genes downstream from spliceosomal mutations.

### Animal models of spliceosomal gene mutations

The study of how spliceosomal alterations affect normal and malignant hematopoiesis will be facilitated by the use of in vivo models that complement primary patient transcriptomes and isogenic cell line models. To this end, conditional knock-in models of the *Srsf2*P95H (Kim et al. 2015; Kon et al. 2015) and *Sf3b1*K700E (Obeng et al. 2014; Mupo et al. 2015) mutations from the endogenous loci of these genes have been published or presented in abstract form. In addition, an inducible transgenic model for mutant *U2af1*S34F expression from the *Col1a1* locus has been published (Shirai et al. 2015).

Despite potential differences in sequence conservation of intronic regions and splicing regulatory elements between species, analyses of the transcriptomes of murine hematopoietic cells expressing mutant *Srsf2*P95H as well as U2af1S34F have already revealed global RNA splicing alterations that are similar to those seen in isogenic human cells and primary patient transcriptomes (Kim et al. 2015; Shirai et al. 2015). Knockout models may prove useful to elucidate the roles of splicing factors in normal hematopoietic development as well as in the context of spliceosomal mutations. For instance, comparison of the hematopoietic effects of conditional deletion of *Srsf2* versus expression of *Srsf2*P95H provided direct evidence that the P95H mutation in *Srsf2* is both biologically and transcriptionally distinct from haploinsufficiency or complete loss of function (Kim et al. 2015).

## Conclusions

Fewer than 5 years have elapsed since the discovery of spliceosomal gene mutations in myeloid malignancies, yet many insights have already been made about the global effects of these mutations on RNA splicing, and initial murine models of several of these mutations have been described. Further rigorous assessment of the mechanistic effects of the mutations on splicing at both genome-wide and locus-specific levels will be critical for future efforts to therapeutically manipulate splicing in spliceosome mutant malignancies. For example, the finding that mutations in *SRSF2* and *U2AF1* alter RNA splicing in a sequence-specific manner may be critically important for developing means to manipulate downstream pathologic splicing mediated by these mutant proteins. Furthermore, although several specific splicing alterations of potential importance have been highlighted by published informatics analyses, systematic functional characterization of these and other potentially important splicing events is needed. This effort will hopefully elucidate novel pathways critical for disease pathogenesis as well as potentially elucidate additional specific therapeutic approaches to target particularly important missplicing events. Finally, the generation of additional murine models bearing genetic alterations mimicking those seen in patients, along with alleles for conditional down-regulation of the same genes, will prove useful for defining splicing regulation throughout both normal and impaired hematopoiesis.

## Supplementary Material

Supplemental Material
