# Supplementary material for: Spliceosomal gene mutations in myelodysplasia: molecular links to clonal abnormalities of hematopoiesis
Source: Genes Dev. 2016 May 1;30(9):989–1001. doi: 10.1101/gad.278424.116 (PMC4863743; doi:10.1101/gad.278424.116)
Supplement: Supplemental Material [file supp_30_9_989__index.html]

Supplemental Material 

# Spliceosomal gene mutations in myelodysplasia: molecular links to clonal abnormalities of hematopoiesis

## Supplemental Material

**Files in this Data Supplement:**

- Supplemental\_Information.doc
