# Supplementary material for: Spliceosomal gene mutations in myelodysplasia: molecular links to clonal abnormalities of hematopoiesis
Source: Genes Dev. 2016 May 1;30(9):989–1001. doi: 10.1101/gad.278424.116 (PMC4863743; doi:10.1101/gad.278424.116)
Supplement: Supplemental Material [file supp_30.9.989_Supplemental_Information.doc]

**Supplemental information**

**References used in Figure 2A and 2B**

Broseus J, Alpermann T, Wulfert M, Florensa Brichs L, Jeromin S, Lippert E, Rozman M, Lifermann F, Grossmann V, Haferlach T et al. 2013. Age, JAK2(V617F) and SF3B1 mutations are the main predicting factors for survival in refractory anaemia with ring sideroblasts and marked thrombocytosis. *Leukemia* **27**: 1826-1831.

Cho YU, Jang S, Seo EJ, Park CJ, Chi HS, Kim DY, Lee JH, Lee JH, Lee KH, Koh KN et al. 2015. Preferential occurrence of spliceosome mutations in acute myeloid leukemia with preceding myelodysplastic syndrome and/or myelodysplasia morphology. *Leuk Lymphoma* **56**: 2301-2308.

Damm F, Kosmider O, Gelsi-Boyer V, Renneville A, Carbuccia N, Hidalgo-Curtis C, Della Valle V, Couronne L, Scourzic L, Chesnais V et al. 2012a. Mutations affecting mRNA splicing define distinct clinical phenotypes and correlate with patient outcome in myelodysplastic syndromes. *Blood* **119**: 3211-3218.

Damm F, Thol F, Kosmider O, Kade S, Loffeld P, Dreyfus F, Stamatoullas-Bastard A, Tanguy-Schmidt A, Beyne-Rauzy O, de Botton S et al. 2012b. SF3B1 mutations in myelodysplastic syndromes: clinical associations and prognostic implications. *Leukemia* **26**: 1137-1140.

Genovese G, Kahler AK, Handsaker RE, Lindberg J, Rose SA, Bakhoum SF, Chambert K, Mick E, Neale BM, Fromer M et al. 2014. Clonal hematopoiesis and blood-cancer risk inferred from blood DNA sequence. *N Engl J Med* **371**: 2477-2487.

Haferlach T, Nagata Y, Grossmann V, Okuno Y, Bacher U, Nagae G, Schnittger S, Sanada M, Kon A, Alpermann T et al. 2014. Landscape of genetic lesions in 944 patients with myelodysplastic syndromes. *Leukemia* **28**: 241-247.

Hahn CN, Scott HS. 2012. Spliceosome mutations in hematopoietic malignancies. *Nat Genet* **44**: 9-10.

Jaiswal S, Fontanillas P, Flannick J, Manning A, Grauman PV, Mar BG, Lindsley RC, Mermel CH, Burtt N, Chavez A et al. 2014. Age-related clonal hematopoiesis associated with adverse outcomes. *N Engl J Med* **371**: 2488-2498.

Kang MG, Kim HR, Seo BY, Lee JH, Choi SY, Kim SH, Shin JH, Suh SP, Ahn JS, Shin MG. 2015. The prognostic impact of mutations in spliceosomal genes for myelodysplastic syndrome patients without ring sideroblasts. *BMC Cancer* **15**: 484.

Lasho TL, Finke CM, Hanson CA, Jimma T, Knudson RA, Ketterling RP, Pardanani A, Tefferi A. 2012a. SF3B1 mutations in primary myelofibrosis: clinical, histopathology and genetic correlates among 155 patients. *Leukemia* **26**: 1135-1137.

Lasho TL, Jimma T, Finke CM, Patnaik M, Hanson CA, Ketterling RP, Pardanani A, Tefferi A. 2012b. SRSF2 mutations in primary myelofibrosis: significant clustering with IDH mutations and independent association with inferior overall and leukemia-free survival. *Blood* **120**: 4168-4171.

Lindsley RC, Mar BG, Mazzola E, Grauman PV, Shareef S, Allen SL, Pigneux A, Wetzler M, Stuart RK, Erba HP et al. 2015. Acute myeloid leukemia ontogeny is defined by distinct somatic mutations. *Blood* **125**: 1367-1376.

Malcovati L, Papaemmanuil E, Bowen DT, Boultwood J, Della Porta MG, Pascutto C, Travaglino E, Groves MJ, Godfrey AL, Ambaglio I et al. 2011. Clinical significance of SF3B1 mutations in myelodysplastic syndromes and myelodysplastic/myeloproliferative neoplasms. *Blood* **118**: 6239-6246.

Meggendorfer M, Roller A, Haferlach T, Eder C, Dicker F, Grossmann V, Kohlmann A, Alpermann T, Yoshida K, Ogawa S et al. 2012. SRSF2 mutations in 275 cases with chronic myelomonocytic leukemia (CMML). *Blood* **120**: 3080-3088.

Nangalia J, Green TR. 2014. The evolving genomic landscape of myeloproliferative neoplasms. *Hematology Am Soc Hematol Educ Program* **2014**: 287-296.

Papaemmanuil E, Cazzola M, Boultwood J, Malcovati L, Vyas P, Bowen D, Pellagatti A, Wainscoat JS, Hellstrom-Lindberg E, Gambacorti-Passerini C et al. 2011. Somatic SF3B1 mutation in myelodysplasia with ring sideroblasts. *N Engl J Med* **365**: 1384-1395.

Papaemmanuil E, Gerstung M, Malcovati L, Tauro S, Gundem G, Van Loo P, Yoon CJ, Ellis P, Wedge DC, Pellagatti A et al. 2013. Clinical and biological implications of driver mutations in myelodysplastic syndromes. *Blood* **122**: 3616-3627; quiz 3699.

Patnaik MM, Lasho TL, Finke CM, Hanson CA, Hodnefield JM, Knudson RA, Ketterling RP, Pardanani A, Tefferi A. 2013. Spliceosome mutations involving SRSF2, SF3B1, and U2AF35 in chronic myelomonocytic leukemia: prevalence, clinical correlates, and prognostic relevance. *Am J Hematol* **88**: 201-206.

Patnaik MM, Lasho TL, Hodnefield JM, Knudson RA, Ketterling RP, Garcia-Manero G, Steensma DP, Pardanani A, Hanson CA, Tefferi A. 2012. SF3B1 mutations are prevalent in myelodysplastic syndromes with ring sideroblasts but do not hold independent prognostic value. *Blood* **119**: 569-572.

Vannucchi AM, Lasho TL, Guglielmelli P, Biamonte F, Pardanani A, Pereira A, Finke C, Score J, Gangat N, Mannarelli C et al. 2013. Mutations and prognosis in primary myelofibrosis. *Leukemia* **27**: 1861-1869.

Visconte V, Makishima H, Jankowska A, Szpurka H, Traina F, Jerez A, O'Keefe C, Rogers HJ, Sekeres MA, Maciejewski JP et al. 2012. SF3B1, a splicing factor is frequently mutated in refractory anemia with ring sideroblasts. *Leukemia* **26**: 542-545.

Wassie E, Finke C, Gangat N, Lasho TL, Pardanani A, Hanson CA, Ketterling RP, Tefferi A. 2015. A compendium of cytogenetic abnormalities in myelofibrosis: molecular and phenotypic correlates in 826 patients. *Br J Haematol* **169**: 71-76.

Yoshida K, Sanada M, Shiraishi Y, Nowak D, Nagata Y, Yamamoto R, Sato Y, Sato-Otsubo A, Kon A, Nagasaki M et al. 2011. Frequent pathway mutations of splicing machinery in myelodysplasia. *Nature* **478**: 64-69.

Zhang SJ, Rampal R, Manshouri T, Patel J, Mensah N, Kayserian A, Hricik T, Heguy A, Hedvat C, Gonen M et al. 2012. Genetic analysis of patients with leukemic transformation of myeloproliferative neoplasms shows recurrent SRSF2 mutations that are associated with adverse outcome. *Blood* **119**: 4480-4485.
